# Upcoming Neurophotonics Status Report

**DOI:** 10.1117/1.NPh.8.4.040101

**Published:** 2021-12-22

**Authors:** Anna Devor, Darcy S. Peterka, Erin M. Buckley, Rickson C. Mesquita

**Affiliations:** a Boston University, Neurophotonics Center, Boston, Massachusetts, United States; b Columbia University, Zuckerman Mind Brain Behavior Institute, New York City, New York, United States; c Georgia Institute of Technology and Emory University, Wallace H. Coulter Department of Biomedical Engineering, Atlanta, Georgia, United States; d Emory University School of Medicine, Department of Pediatrics, Atlanta, Georgia, United States; e University of Campinas, Institute of Physics, Campinas, São Paulo, Brazil

## Abstract

Forthcoming status report articles provide updates on microscopy and on diffuse optical imaging in neurophotonics.

Do you get this feeling that it is hard to keep up with all the developments in neurophotonics, with new tools and methods popping up faster than bread in a toaster? We know the feeling! Last spring, we decided to engage our community and produce a pair of articles featuring the “latest and greatest” photonics technologies that are targeted to neuroscience. The first article focuses on tools that are, in general, applicable to animal models and usually considered under the umbrella of microscopy. In practice, however, these tools span from the nanoscale of molecular sensors to the mesoscale of cortical columns and entire brain areas (hundreds of microns). The second article focuses on diffuse optical imaging methods that are applicable to humans and describes any technique that employs a near-infrared light source and a photodetector to capture multiply scattered photons in the diffusion limit. Each one is a snapshot of the present moment, a “status report.”

**Figure:**
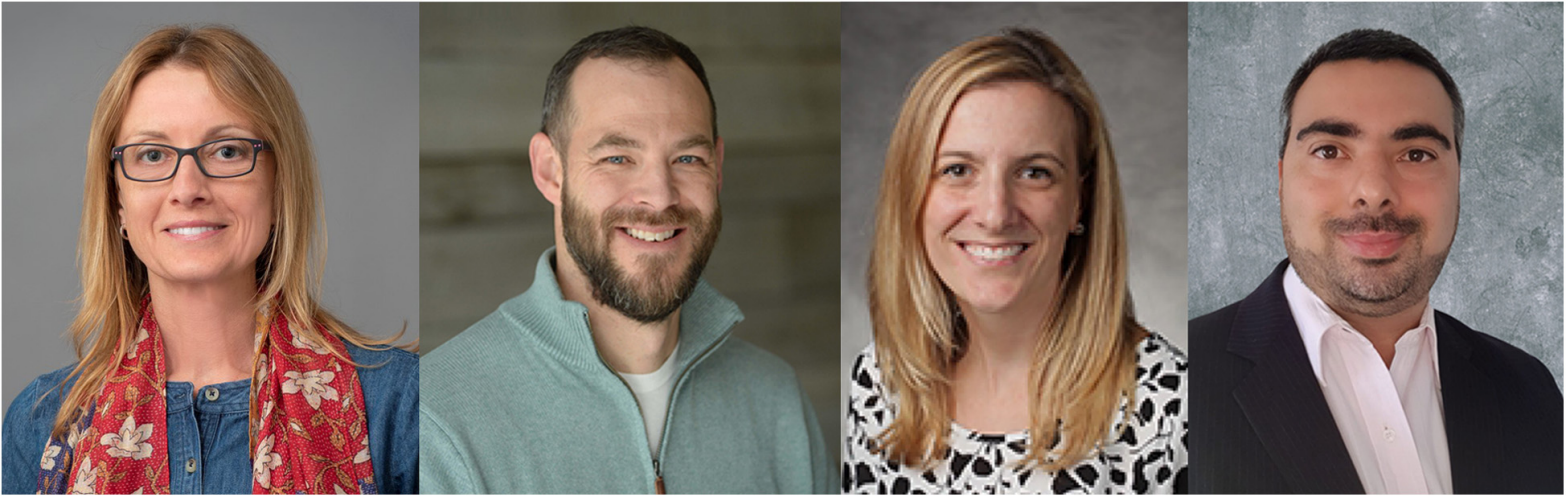
From left to right: Anna Devor (Boston Univ.), Darcy S. Petarka (Columbia Univ.), Erin M. Buckley (Georgia Institute of Technology and Emory Univ.), and Rickson C. Mesquita (Univ. of Campinas).

**Figure:**
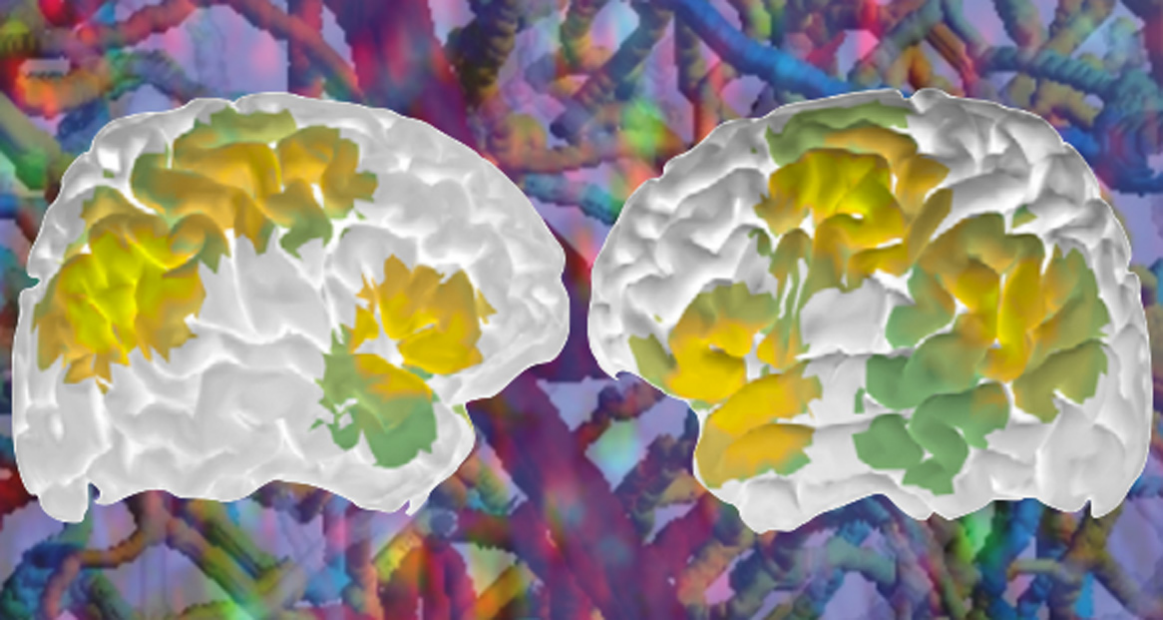
Artistic rendering of the concept of imaging the brain across scales. Task-induced human brain activity obtained with functional near-infrared spectroscopy (fNIRS; image from Novi et al., doi 10.1117/1.NPh.7.1.015001) is overlaid on microscopically resolved brain cells and blood vessels.

In the last decade, in the world of microscopic neurophotonics, we have seen remarkable progress in many areas, including the molecular engineering of optical probes, high-throughput structural and molecularly annotated imaging in cleared blocks of brain tissue. Labs are imaging deeper than before *in vivo*, with 3-photon excitation, and there has been an explosion of strategies for sculpting and controlling light for increasing temporal resolution, photoactivation, and extended field-of-view imaging. These methods are also coupled with new microscopes that allow either extremely high-speed imaging, large fields of view, or both.

Similarly, the world of diffuse optical imaging has seen tremendous progress in static and dynamic near-infrared spectroscopy (NIRS) techniques, ranging from advances in miniaturization, wearability, cost reductions, and depth penetration. The advances in hardware have opened doors to explore unanswered questions in several fields, ranging from neurodevelopment to social and cognitive sciences. The bedside monitoring capabilities have led to numerous clinical and global health applications. The increasing demand for these techniques in challenging situations has driven the appearance of novel data analysis methodologies and algorithms that improve accuracy, depth penetration, and spatial sensitivity.

After months of labor and the substantial efforts of many in the community, we are happy to announce that both status report articles are coming out in early 2022. Due to their considerable size—reflecting the breadth of recent neurophotonic developments—each article will appear as a stand-alone issue of *Neurophotonics*. Follow us on social media or sign up to receive *Neurophotonics* updates to be among the first to read it! We would also like to take this opportunity to thank all the contributing authors for their enthusiastic support of the status report project!

Finally, this is the last editorial for 2021. On behalf of the entire *Neurophotonics* crew, we wish you Happy New Year! Let it be one more year of global science, inspiration, and discovery!

